# What is the patient acceptance when only scapulectomy is possible in case of malignant tumor? A case series

**DOI:** 10.1016/j.jseint.2022.08.005

**Published:** 2022-09-08

**Authors:** Hugo Barret, Olivier Bozon, Guillaume Fassot, Michel Chammas, Bertrand Coulet, Cyril Lazerges

**Affiliations:** a Hand and Upper Limb Surgery Department, Lapeyronie University Hospital, Montpellier, France; b Trauamtology-Orthopaedics and Plastic-Reconstructive Surgery Department, CHU Gabriel Montpied, Clermont-Ferrand, France

**Keywords:** Partial or total scapulectomy, Mental health, Quality of life, Malignant tumor, Functional outcome of the scapulectomies, Emotional acceptance

## Abstract

**Background:**

Scapulectomy is one of the surgical options in the case of malignant lesions in the scapula with an indication of surgical removal. Very few series in the literature have looked at postoperative quality of life and emotional acceptance, particularly in the case of scapulectomy without reconstruction. The objective is to assess the midterm results of scapulectomies in terms of function, quality of life, and acceptance for the patient.

**Methods:**

With a mean follow-up of 85 months (range 42 months-180 months), 11 scapulectomies for malignant tumors were performed with a mean age of 50 years: 5 partial scapulectomies, 4 total scapulectomies, and 2 subtotal scapulectomies. There were 6 chondrosarcomas, 2 high-grade osteosarcomas, 1 malignant peripheral nerve sheath tumor, and 1 low-grade atypical epithelioid sarcoma. The radio-clinical analysis was focused on functional results and mental health evaluation.

**Results:**

The mean Musculoskeletal Tumor Society score of 11 scapulectomies was 20 ± 5 at 66% of normal, with the Disabilities of the Arm, Shoulder and Hand (DASH) score of 35 ± 26, and the Toronto Extremity Salvage Score of 76%. Patients had controlled pain (mean visual analog scale 1/10). Mobilities of the 11 scapulectomies were correct: average active anterior elevation of 89.5 ± 43 degrees, average abduction of 81 ± 42 degrees, average external rotation of 30 ± 25 degrees, and average internal rotation was at L5. Scapulectomy results in impaired physical and mental health compared with the general population (PCS-12 = -9; MCS-12 = -7). Partial scapulectomy, compared to total scapulectomy, gave better results: Musculoskeletal Tumor Society score (14 ± 1 vs. 24 ± 1 *P* = .0175), acceptance (45 ± 9 vs. 92 ± 16, *P* = .0184), mental health (MCS-12: 29 ± 1 vs. 55 ± 4, *P* = .0175), and Toronto Extremity Salvage Score (84 ± 5 vs. 68 ± 7, *P* = .0195). Partial and subtotal scapulectomies were better accepted (45 ± 9 vs. 86 ± 23, *P* = .0323) and tolerated (MCS-12: 29 ± 1 vs. 52 ± 6, *P* = .0099) by the patient compared to total scapulectomy.

**Conclusion:**

Total or partial scapulectomies without scapula reconstruction remain a disabling procedure performed with consequences on the physical and mental health of the patients. Partial or subtotal scapulectomy should be performed whenever possible because it seems to be associated with a better functional prognosis as well as less poor mental health and emotional acceptance of the patients, even though total scapulectomy may be necessary to obtain a complete curative tumor resection, which is the main goal.

Scapular localization of malignant lesions is classically encountered in oncology: chondrosarcomas are located in 20% of cases at the level of the scapular girdle.^21^ Curative treatment of a malignant lesion of the scapula always consists of partial or total “en bloc” resection of the scapula, which may be associated with bony or prosthetic reconstruction.^31^ Scapulectomy allows preservation of the upper limb with variable functional results, according to the few series available in the literature.^8^^,^^10^^,^^11^^,^^15^^,^^17^^,^^18^^,^^23^^,^^24^^,^^30^^,^^31^ Partial (preservation of the glenohumeral joint) and subtotal (ie, with preservation of the acromion or part of the scapula) scapulectomies have shown a positive functional outcome and appear to be superior to total scapulectomies.^10^^,^^13^ Some studies, such as Nota's,^21^ analyzing factors reducing survival, have shown the poor prognosis of metastases, recurrent tumors, or high-grade tumors during this surgery. As we have seen, the first step in a malignant lesion of the scapula is scapulectomy. Once performed, it may be accompanied by reconstruction of the scapula or the glenohumeral joint. All surgical options for reconstruction by arthroplasty or bone allograft require a certain number of local criteria: preservation of essential muscles such as the deltoid,^25^ trapezius, rhomboids,^22^ one of the rotator cuff muscles^15^ or the acromion.^10^ However, when local conditions require aggressive resection of the peri scapular soft tissues with the impossibility of preserving the trapezius, deltoid, or latissimus dorsi muscle, the only solution is isolated scapulectomy^27^ without reconstruction. Very few series in the literature have looked at postoperative quality of life and emotional acceptance after isolated scapulectomy.^30^ Once the planning of the tumor resection is established, the precise analysis, with a referenced scale, of the quality of life, acceptance, and functional outcome of the scapulectomies seems important for the surgery and the further life of the operated patients. Therefore, we conducted a retrospective study analyzing the results of scapulectomies on the mental health of patients in the medium term of follow-up, in particular their quality of life and acceptance. The primary objective of our study is to know the medium-term results of scapulectomies on the quality of life and acceptance of patients. The secondary objective is to know the functional clinical results as well as the complications and recurrences. The hypothesis of our study is that partial resections are better accepted by the patient than total resections.

## Materials and methods

### Patients

Between January 2002 and August 2015, we included in our study all patients who underwent total, subtotal, or partial scapulectomy in our center. All patients presented a (1) primary malignant tumor of the scapula, with criteria of local aggressiveness, leading to (2) invasion of the soft tissues or malignant soft tissue tumors with bone extension of more than 50% of the scapula (3) and accepted a (4) prolonged follow-up. Metastatic lesions and treatments with scapula curettage or glenohumeral prostheses were excluded. The patients were informed of the study and all gave their consent. The study was approved by the ethics committee of the Hospital.

**Figure 1:**
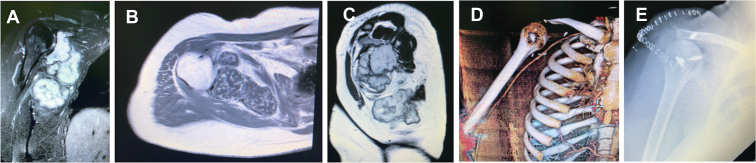
Example of total scapulectomy with suspensoplasty. Tumor invading the entire scapula: frontal MRI section (T2 STIR, **A**) with hyper signal lesion in the scapula, axial section (T1, **B**) with lesion of the body of the scapula in hyposignal and sagittal section (T1, **C**) with hyper signal lesion. Resection of the entire scapula visualized on the 3D CT scan (**D**) and postoperative radiograph (**E**) associated with resection of the trapezius, deltoid and posterior superior cuff and suspension with anchors. *MRI*, magnetic resonance imaging; *3D*, 3-dimensional; *CT*, computed tomography.

Eleven scapulectomies in 11 patients were performed: 5 partial scapulectomies, 4 total scapulectomies (Fig. 1), and 2 subtotal scapulectomies. There were 2 grade 1 chondrosarcomas, 4 grade 2 chondrosarcomas, 1 grade 3 chondrosarcoma, 2 high-grade osteosarcomas, 1 malignant peripheral nerve sheath tumor, and 1 low-grade atypical epithelioid sarcoma. One patient had preoperative chemotherapy (methotrexate), 4 patients had postoperative chemotherapy (2 patients with methotrexate and 2 patients with doxorubicin), and 1 patient had postoperative radiotherapy. The mean follow-up was 85 months (range 42 months-180 months) and the mean age of patients was 50 years old (25-77 years old).

### Surgical technique

All patients were operated on by the same senior surgeon and underwent a surgical biopsy prior to the scapulectomy, sent to anatomopathology. The installation was done in all cases in lateral decubitus controlateral to the scapulectomy. The scar from the biopsy was to be systematically taken into the S-shape approach. The dissection was done with sufficient margin, using en bloc resection and trying to be as conservative as possible on the peri scapular muscles. The surgical specimen was sent to the pathology department and the closure was performed on a drain.

### Postoperatively

Patients were immobilized with an elbow sling for 6 weeks. Early mobilization of the elbow and fingers was performed. The first follow-up visit allowed verification of the resection margins and of the good wound healing.

### Clinical evaluation

In this continuous single-operator series, all patients were evaluated clinically at the last follow-up by an independent observer of the surgeon. We assessed pain using the visual analog scale (VAS: 0 being no pain and 10 being unbearable pain). Mobility of the shoulder, elbow, and wrist was assessed with a standard goniometer. Physical function was assessed with the Musculoskeletal Tumor Society (MSTS) score^5^ which includes 6 items scored from 0 to 5: pain, function, hand position, dexterity, lifting ability, and emotion^6^. We assessed the function of the upper limb with the Disabilities of the Arm, Shoulder and Hand (DASH) score^12^ and the quality of life with the SF 12.^7^ The SF 12 allows an assessment of physical and mental health, a crucial point in our study with a comparison to European standards. Subjective assessment of the shoulder was performed with the Subjective Shoulder Value score.^9^ We also assess emotional acceptance, which is a self-rated function of the patient's perception of the overall outcome within the MSTS score. We also evaluated the impact on capacity and potential disability after scapulectomy via the Toronto Extremity Salvage Score (TESS).^3^^,^^4^

### Radiological assessment

Preoperatively, we used the Malawer classification in 6 stages^16^ separated in 2 categories with intra or extra articular resection as well as the JMOG classification which separates scapulectomies in 5 stages.^11^ We divided the series into 3 groups according to the type of scapulectomy performed^30^: partial (glenohumeral joint conserved), subtotal (ie, with conservation of the acromion or part of the scapula), and total scapulectomy. Follow-up was performed with shoulder x-rays as well as magnetic resonance imaging, computed tomography scans of the chest and shoulder annually in oncology and orthopedics.

### Statistical analysis

Means and standard deviation were calculated for continuous variables. Quantitative variables (MSTS score, DASH score, mobilities and pain) were analyzed by the Mann. The value of *P* < .05 was considered to be statistically significant. Easy med stat software was used.

## Results

The characteristics of the patient, the lesion, and the type of resection are listed in Table I.

**Table I:** Population.

Patients	Gender	Age	Side	Dominant side operated	Type of lesion	Type of intervention	Malawer classifcation	JMOG classification	Associated procedure	Associated muscle resection
1	F	56	L	N	Grade 1 chondrosarcoma	Partial scapulectomy	3	2	Postoperative chemotherapy with doxorubicin and postoperative radiotherapy	Resection of the deltoid and trapezius muscles
2	M	58	L	Y	Grade 2 chondrosarcoma	Partial scapulectomy	2	4	None	Resection of the supraspinatus, infraspinatus, subscapularis, rhomboids, anterior serratus, and trapezius muscles
3	M	25	R	Y	Grade 1 chondrosarcoma	Partial scapulectomy	2	4	None	Resection of the supraspinatus, infraspinatus, subscapularis, rhomboids, and latissimus dorsi muscles
4	F	61	L	Y	Osteolipoma	Partial scapulectomy	2	5	None	Resection of the infraspinatus and latissimus dorsi muscles
5	M	46	R	N	Neurofibrosarcoma	Subtotal scapulectomy	2	4	Humeral suspensoplasty Postoperative chemotherapy with doxorubicin	Resection of the supraspinatus, infraspinatus, subscapularis, anterior serratus, and trapezius muscles
6	M	77	R	Y	Grade 2 chondrosarcoma	Subtotal scapulectomy	3	3	Humeral suspensoplasty	Resection of the supraspinatus, infraspinatus, latissimus dorsi, rhomboids, anterior serratus, and trapezius muscles
7	F	25	R	Y	Grade 2 chondrosarcoma	Total scapulectomy	1	3	Humeral suspensoplasty	Resection of the supraspinatus, infraspinatus, subscapularis, latissimus dorsi, rhomboids, anterior serratus, and trapezius muscles
8	M	40	R	Y	Grade 3 chondrosarcoma	Partial scapulectomy	2	5	None	Resection of the infraspinatus, subscapularis, latissimus dorsi, rhomboids, anterior serratus, and trapezius muscles
9	M	70	L	N	Atypical epitheloid sarcoma	Total scapulectomy	6	1	Humeral suspensoplasty	Resection of the supraspinatus, infraspinatus, subscapularis, latissimus dorsi, rhomboids, anterior serratus, and trapezius muscles
10	M	19	L	Y	High-grade osteosarcoma	Total scapulectomy	6	1	Humeral suspensoplasty Preoperative and postoperative chemotherapy with methotrexate	Resection of the supraspinatus, infraspinatus, subscapularis, latissimus dorsi, rhomboids, anterior serratus, and trapezius muscles
11	F	72	L	N	High-grade osteosarcoma	Total scapulectomy	5	1	Humeral suspensoplasty Postoperative chemotherapy with methotrexate	Resection of the supraspinatus, infraspinatus, and subscapularis, muscles

*JMOG*, Japanese Musculoskeletal Oncology Group; *F*, female; *M*, male; *L*, left; *R*, right; *N*, No; *Y*, Yes.

### Mental health outcomes

The mean MCS 12 score for the series reflecting mental health was 43 ± 12, 7 points below the average in the European population (Table II). Acceptance was 71% ± 27. Regarding physical health, the PCS 12 score is 9 points lower than the general European population. The patient's subjective assessment (Subjective Shoulder Value score) of his shoulder is slightly higher than 50% of a normal shoulder.

**Table II:** Subjective functional and quality of life evaluation after scapulectomy.

	Average ( ± DS)
SSV Score	55 ± 29
Acceptation%	71 ± 27
PCS-12	41 ± 7
Difference from the average European population	−9
MCS-12	43 ± 12
Difference from the average European population	−7

*SSV*, Subjective Shoulder Value score; *PCS*, physical component score; *MCS*, mental component score.

### Clinical results

The mean mobilities in the whole series were in active anterior elevation 89.5 ± 43 degrees, abduction 81 ± degrees, and external rotation 30 ± 25 degrees. The mean internal rotation was at the level of L5. For all patients, elbow and wrist mobilities were normal. As presented in Table III, the mean MSTS score was 20 ± 5 at 66% of normal with a mean DASH score of 35 ± 26. Pain was rated at 1/10 on the visual analog scale on average. The mean TESS score was 76 ± 9.

**Table III:** Functional evaluation scores of the operated upper limb.

	Average ( ± DS)
MSTS Pain	4.5 ± 0.6
MSTS fonction	2.4 ± 1.3
MSTS hand positionning	3.2 ± 0.9
MSTS manual dexterity	3.5 ± 0.9
MSTS lifting ability	2.7 ± 1.8
MSTS emotional	3.5 ± 1.4
Total MSTS	20 ± 5
% MSTS	67 ± 18
DASH score	35 ± 26

*MSTS*, Musculoskeletal Tumor Society score; *DASH*, Disabilities of the Arm Shoulder and Hand score.

**Figure 2:**
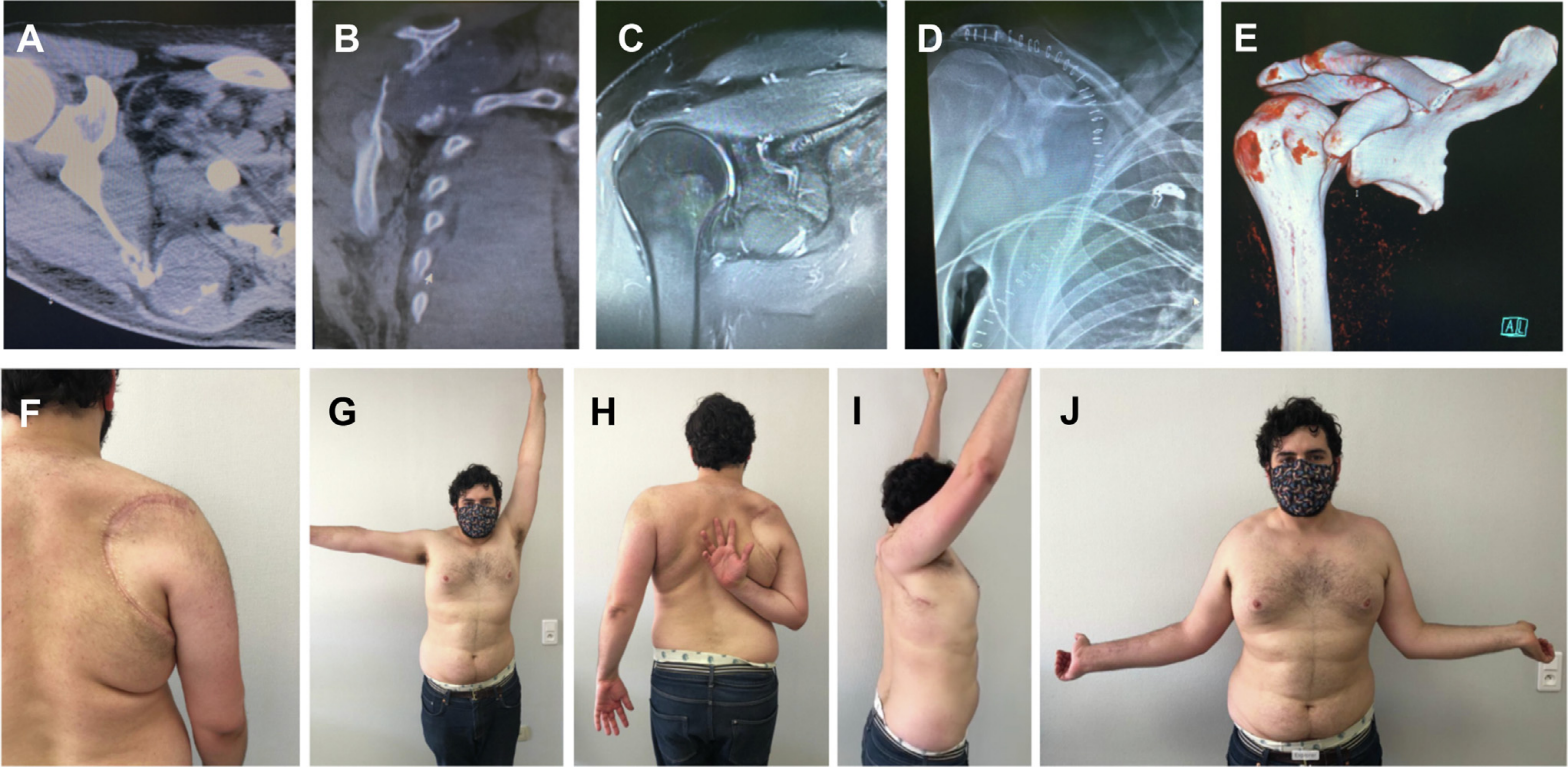
Example of partial scapulectomy. Possible glenohumeral conservation following a lesion of the body of the scapula seen in axial (**A**) and sagittal section on CT scan (**B**) as well as on frontal sections on MRI (T1, **C**) resection of the body of the scapula, the rhomboid and latissimus dorsi muscles. Radiological (**D**) and CT scan (**E**) findings postoperatively with the scar (**F**). At 7 years postoperative, here are the clinical results of this 31-year-old patient: abduction mobility (**G**), internal rotation (**H**), active anterior elevation (**I**), and external rotation (**J**). *MRI*, magnetic resonance imaging; *CT*, computed tomography.

Comparison of clinical results and emotional acceptance between the different types of scapulectomy:

Clinical results and emotional acceptance were better with partial scapulectomy (Fig. 2) than with total scapulectomy (Table IV). Partial and subtotal scapulectomies are better accepted and tolerated by the patient (Table V).

**Table IV:** Comparison between total scapulectomy versus partial scapulectomy.

	Total scapulectomy (n = 4)	Partial scapulectomy (n = 5)	*P*
AAE	41 ± 12	118 ± 25	.0189
Abduction	41 ± 12	112 ± 22	.0189
ER1	5 ± 9	55 ± 13	.0175
IR	buttom	L2	.02
SSV score	21 ± 11	77 ± 15	.0189
DASH score	61 ± 7	11 ± 6	.0195
Total MSTS	14 ± 1	24 ± 1	.0175
% MSTS	46 ± 4	81 ± 5	.0175
Acceptation	45 ± 9	92 ± 16	.0184
PCS-12	38 ± 2	43 ± 8	.2168
MCS-12	29 ± 1	55 ± 4	.0175
TESS Score	68 ± 7	84 ± 5	.0195

*AAE*, active anterior elevation; *ER1*, external rotation elbow against the body; *IR*, internal rotation; SSV *Score*, Subjective Shoulder Value Score; *DASH Score*, The Disabilities of the Arm, Shoulder and Hand score; *MSTS*, Musculoskeletal Tumor Society scoring system; *PCS*, physical component score of SF 12 score (Short Form Health Survey); *MCS*, mental component score of SF 12 score (Short Form Health Survey); *TESS Score*, Toronto Extremity Salvage Score.

**Table V:** Comparison between total scapulectomy versus partial and subototal scapulectomy.

	Total scapulectomy (n = 4)	Partial and subtotal scapulectomy (n = 7)	*P*
AAE	41 ± 12	117 ± 28	.0102
Abduction	41 ± 12	104 ± 23	.0099
ER1	5 ± 9	45 ± 19	.0159
IR	buttom	L5	.07273
SSV score	21 ± 11	75 ± 14	.0104
DASH score	61 ± 7	20 ± 21	.0462
Total MSTS	14 ± 1	24 ± 3	.0097
% MSTS	46 ± 4	79 ± 10	.0097
Acceptation	45 ± 9	86 ± 23	.0323
PCS-12	38 ± 2	44 ± 7	.1042
MCS-12	28 ± 1	52 ± 6	.0099
TESS Score	68 ± 7	81 ± 6	.029

*AAE*, active anterior elevation; *ER1*, external rotation elbow against the body; *IR*, internal rotation; *SSV Score*, Subjective Shoulder Value Score; *DASH Score*, The Disabilities of the Arm, Shoulder and Hand score; *MSTS*, Musculoskeletal Tumor Society scoring system; *PCS*, physical component score of SF 12 score (Short Form Health Survey); *MCS*, mental component score of SF 12 score (Short Form Health Survey); *TESS Score*, Toronto Extremity Salvage Score.

### Complications and recurrences

Two patients presented with early hematomas that were evacuated in the operating room with the initiation of antibiotic therapy. One patient presented Morel-Lavallé syndrome treated by iterative function and then by surgical revision for padding with a favorable evolution. One patient had an area of skin necrosis treated by local care with a good evolution. The analysis of the specimens at the pathology laboratory showed complete resection with adequate margin. No local recurrence in the series was noted. Two patients died due to metastases and we took their results at the last follow-up.

## Discussion

In the case of malignant tumors, scapulectomy, whether partial or total, allows the salvage of the upper limb concerned.^31^ It seems to alter the function of the upper limb and shoulder despite better results with partial scapulectomy and muscle preservation.^15^ In our study, scapulectomy, whether partial or total, leads to an alteration in physical and mental health compared to the general European population, with scores 7 points lower on mental health assessed by the SF 12 and 9 points lower on physical health. Mental health, acceptance, and quality of life were better with partial or subtotal scapulectomy compared to total scapulectomy. The functional outcomes of our series are favorable with an active anterior elevation around the horizontal and rotations allowing the activities of daily life. Partial scapulectomy gave better objective functional results on range of motion.

There are few studies (Table VI) analyzing the mental health and quality of life after partial or total exclusive scapulectomies with a precise score such as SF 12 during a midterm follow-up. Schwab et al^25^ showed us that emotional acceptance and quality of life did not differ if there was or not a deltoid lesion or axillary nerve lesion. In their series of 27 patients with 20 partial scapulectomies, Klein et al^15^ found a correct emotional acceptance of scapulectomy evaluated by the MSTS score or the TESS score. In this series, we found very few details on patient mental health and quality of life after the different types of scapular surgery. Gibbons et al^8^ in their study analyzing the results of subtotal scapulectomies found satisfactory acceptance assessed by the MSTS score with an average of over 80%. These results are in agreement with the study by M. Vahanan et al^17^ who, despite two-thirds of total scapulectomies, found satisfactory acceptance in more than 80% of patients. Thanks to our precise evaluation of the quality of life as well as mental and physical health, our study shows that the more the bone stock is spared with partial or subtotal scapulectomies, the less the mental health will be altered. This result encourages us in the maximum bone and muscle preservation during scapulectomies in order to guarantee the best quality of life. This must be compatible with carcinologic efficiency, “en bloc resection” and the absence of local recurrence in all the cases as for all the patients in our study.

**Table VI:** Summary of the different studies in the literature on the functional clinical results after scapulectomies and on the quality of life.

Authors	Type of procedure	Nb of pt	Mean age	Malignant tumors	FU (m)	Oncologic Efficacy	Complications	MSTS score	Acceptance (MSTS)	TESS score	SF 12	Com
Kiss et al.^14^	- 7 partial S. - 13 total S.	20	42	all	56	14% of recurrence	- 0%	- 88% partial S. - 65% Total S.	- 4 (partial S.) - 3.71 (total S.)	/	/	
Klein et al.^15^	- 7 partial S. - 20 total S.	27	46	- 22 malignant tumor - 5 benign tumor	71	17% of recurrence	- 0%	83.3		- 83% partial S. - 63.8% total S.		Better function with SS
Gibbons et al.^8^	- Subtotal S.	14	42	All	52	14% of recurrence	- 29% - 1 wound infection - 3 considerable pain (2 brachial neuropathy)	71.6	4.3	79.9	/	Preservation of the humeral glenoid joint Reinsertion of the deltoid
Puchner et al.^23^	- 7 subtotal S. - 8 total S - 7TLP - Total resection + endo prothesis	29	40	All	60	- 47% survival at 5-yr follow-up - 3 (13%) local reccurence	34% - 2 infection - 4 Morel-Lavallé - 2 radial nerve lesion - 1 thrombosis - 1 dislocation of prosthesis	- 69% in total - 76% subtotal S. - 68% total S. - 58% TLP	/	/	/	
Vahanan et al.^17^	- 8 partial S. - 15 total S.	23	29	19	67	- 76% survival rate at 5-yr follow-up	13% - 2 infection - 1 cutaneous necrosis	/	83% acceptable result	/	/	Better outcome of subtotal scapulectomy
Griffin et al.^10^	- 16 partial S. - 8 total S.	24	44	All (chondro sarcoma)	81	- 88% survival rate at 10-yr follow up	12.5% - 2 infection - 1 fracture on TLP	84% 90% partial S. vs. 68% total S.	/	87% 96% partial S. vs. 70% total S.	/	
Mimata et al.^18^	- 3 partial S. - 2 subtotal S. - 3 total S.	8	49	All (primary or metastatic sarcomas)	55	- 62.5% survival rate at 5-yr follow up	0	84% 97% partial S. 77% subtotal S. 62% total S.	4.25 5 partial S. 4.5 subtotal S. 3.3 total S.	/	/	latissimus dorsi: stabilizers of the proximal humerus after scapulectomy.
Hayashi et al.^11^	- 22 partial/subtotal S. - 26 total S.	48	46	All	62	/	/	70% 63% total S.	3.7 3.2 total S.	/	/	
Our Study	- 5 partial S. - 2 subtotal S. - 4 total S.	11	50	All	85	- 81% survival rate at 5-yr follow-up	36% - 2 hematomas - 1 Morel Lavallé syndrom - 1 skin necrosis	66% 81% partial S. 71% subtotal S. 46% total S	3.55	76% 84% partial S. 75% subtotal S. 68% total S.	PCS 12 = 41 MCS 12 = 43	/

*FU*, follow-up; *Nb of pt*, number of patient; *MSTS*, Musculoskeletal Tumor Society; *TESS*, Toronto Extremity Salvage Score; *SF*, short form health survey; *S*, scapulectomy; *SS*, supraspinatus; *TLP*, Tikhoff-Linberg procedure; *PCS*, physical component score; *MCS*, mental component score.

From a clinical and functional point of view, MSTS score of Klein study is better than that of our series (25 points versus 20 points). But it is composed of a large majority of partial scapulectomies. The TESS score is comparable to the results of our series for partial (83% versus 84%) or total (68% versus 64%) scapulectomies. Although there is an alteration of the upper limb for all types of scapulectomies, partial scapulectomy has better results in the study of Klein et al.^15^ Preservation of the supraspinatus tendon improves the functional result while the benign or malignant character of the tumor does not modify the result. This notion of cuff preservation is contrary to the series of 14 subtotal scapulectomies by Gibbons et al^8^ with glenohumeral preservation, which showed us that there was no impact of cuff removal if the deltoid and trapezius were preserved and sutured. The series of Gibbons et al had a shorter follow-up than our study (52 months versus 85 months) with a higher MSTS score (72% versus 66%). Three patients had very significant pain of neuropathic origin, which was not found in our series. In their series of 29 cases of scapulectomy with only 2 reconstruction prostheses, Puchner et al^23^ found functional results equivalent to our series in terms of the MSTS score. The more invasive the procedure with extensive bone resection, the lower the MSTS score in both the Puchner study and our study. In a short-term follow-up, Vahanan et al^17^ found a better functional result with scapulectomy with glenohumeral conservation, which confirms the results of our study. A series of 7 patients with scapulectomy^11^ including 4 patients who had stabilization with soft tissue reconstruction found a correct function of the upper limb thanks to the mobility of the elbow, wrist, and fingers. On the other hand, no difference in functional outcome between the group with and without reconstruction. However, Mimata et al^18^ have shown the importance of preservation of the latissimus dorsi for the functional prognosis after scapulectomy, again without taking into account the mental impact. Min et al^19^ proposed the use of a scapular hemiarthroplasty to preserve the humeral bone stock. The postoperative mobilities are not better than those of our study despite the use of artificial ligament which allows a better functional score.

Few studies have compared the differences in emotional acceptance and quality of life between the different types of scapulectomy. In the study by Mimata et al,^18^ emotional acceptance is excellent with partial scapulectomy and decreases with greater bone resection. Hayashi et al^11^ confirm their results and those of our study with a lower acceptance with total scapulectomy. Kiss et al^14^ in their heterogeneous study on shoulder resections for musculoskeletal tumors found similar results to our study with a better quality of life for the partial scapulectomy group.

Partial or total scapulectomy allows oncological control, especially for chondrosarcomas, with a very low rate of local recurrence and metastases as shown by Griffin et al.^10^ These results are comparable to our series which shows no local recurrence. The functional results in the series by Capanna et al^2^ analyzing allograft reconstructions after scapulectomy are no better than those in our series. The complication rate in this study by Capanna is high (66% complications and 33% revisions) due to allograft fractures or material breakage. Ayvaz et al^1^ also showed that scapulohumeral constrained prostheses had a higher than expected complication rate with a short lifespan (about 20% revisions at less than 3 years of follow-up). Total or partial scapulectomy in our study has an acceptable rate of complications which are most often treated without reoperations with a satisfactory clinical evolution and acceptance.

Our study has several limitations since it is a retrospective study with a small number of patients. This is a historical series in a reference center for bone and soft tissue cancers, including exclusively scapulectomies, which explains the small number of patients. On the other hand, this is a homogeneous monocentric study using a single method, namely scapulectomy without reconstruction by prosthesis^20^^,^^26^^,^^28^^,^^29^ or by allograft^32^ of the scapula. This approach was justified by the fact that each of the patients included in this series required extensive soft tissue resection, most often involving the rotator cuff muscles, deltoid, trapezius, latissimus dorsi, and rhomboids muscle. As previously mentioned, in these extensive resection conditions, prostheses or allograft do not provide better results than scapulectomy. This study has several strong points since it is a surgery performed by the same surgeon and an average follow-up of more than 7 years. It analyzes the functional result but also the result on the mental health of this disfiguring surgery and the emotional acceptance by the patient. However, we cannot establish causality in this retrospective study. There appears to be an association between the degree of resection and mental health. However, this association may be biased by a worse prognosis and a more difficult oncological situation in the total scapulectomy group

## Conclusion

Total or partial scapulectomy without reconstruction of the scapula remains a disabling procedure performed in cases where no other surgery is possible. It allows saving the upper limb. It is not without consequences on the physical and mental health of the patients. Partial or subtotal scapulectomy should be performed whenever possible because it seems to be associated with a better functional prognosis as well as less poor mental health and emotional acceptance of the patients, even though total scapulectomy may be necessary to obtain a complete curative tumor resection, which is the main goal.

## Disclaimers:

Funding: No funding was disclosed by the authors.

Conflicts of interest: The authors, their immediate families, and any research foundation with which they are affiliated have not received any financial payments or other benefits from any commercial entity related to the subject of this article.
